# Children's Separation Anxiety Scale (CSAS): Psychometric Properties

**DOI:** 10.1371/journal.pone.0103212

**Published:** 2014-07-29

**Authors:** Xavier Méndez, José P. Espada, Mireia Orgilés, Luis M. Llavona, José M. García-Fernández

**Affiliations:** 1 Department of Personality, Psychological Assessment and Treatment, University of Murcia, Murcia, Spain; 2 Department of Health Psychology, Miguel Hernández University, Elche, Spain; 3 Department of Personality, Psychological Assessment and Treatment (Clinical Psychology), Complutense University, Madrid, Spain; 4 Department of Developmental Psychology and Teaching, University of Alicante, Alicante, Spain; Erasmus University Rotterdam, Netherlands

## Abstract

This study describes the psychometric properties of the Children's Separation Anxiety Scale (CSAS), which assesses separation anxiety symptoms in childhood. Participants in Study 1 were 1,908 schoolchildren aged between 8 and 11. Exploratory factor analysis identified four factors: worry about separation, distress from separation, opposition to separation, and calm at separation, which explained 46.91% of the variance. In Study 2, 6,016 children aged 8–11 participated. The factor model in Study 1 was validated by confirmatory factor analysis. The internal consistency (α = 0.82) and temporal stability (*r* = 0.83) of the instrument were good. The convergent and discriminant validity were evaluated by means of correlations with other measures of separation anxiety, childhood anxiety, depression and anger. Sensitivity of the scale was 85% and its specificity, 95%. The results support the reliability and validity of the CSAS.

## Introduction

Separation anxiety disorder (SAD) in children is characterized by excessive and inappropriate anxiety for the child's stage of development, and which he or she experiences on being separated from attachment figures – generally the parents – or spending time outside his or her home [1]. This disproportionate anxiety manifests itself in distress, worry and resistance to or rejection of the separation. Prevalence of SAD is 3.9% in childhood (6–12 years) and 2.6% in adolescence (13–18 years), according to two meta-analyses carried out with 13 and 26 epidemiological studies, respectively [2]. SAD and some types of specific phobia, such as those related to animals, are the anxiety disorders with earliest age of onset, the majority of cases emerging prior to age 12 [3]. The presence of SAD in childhood predicts this same disorder in adolescence (age 13–19) [4]. SAD is a strong risk factor (78.6%) for the development of psychopathology in young adulthood (age 19–30), so that the diagnosis, assessment and treatment of children with SAD are relevant for preventing the appearance of disorders such as panic and depression [5].

Clinical diagnosis and assessment aspire to collect as much information as possible at the least possible cost in time and money, and in effort for the child, parents and professionals. Optimization of assessment efficiency involves the avoidance of extreme positions. On the one hand, excessively thorough assessment leads to fatigue and to the risk of loss of precision and early abandonment of the therapeutic relation. On the other, very brief assessment is of little use for planning treatment, and involves risks such as the omission of relevant data and premature therapeutic decisions. In the framework of multi-method assessment, self-report rating scales are widely used, together with structured interviews, as they are easy to apply, fill out and evaluate. They are especially useful from the age of 7 onwards, when the child has acquired sufficient reading ability and self-assessment skills. There are general self-reports for assessing the different childhood anxiety disorders, including SAD, such as the Screen for Child Anxiety Related Emotional Disorders (SCARED) [6] or the Spence Children's Anxiety Scale (SCAS) [7]. The comprehensive application of these instruments, which include more than 40 items, is of great use for screening in epidemiological studies, but may be unnecessary and excessive in clinical cases of SAD, detected via interview; at the same time, application of just the SAD scales, with their less than 10 items, may be insufficient for assessing the full spectrum of symptoms and drawing up a treatment plan. Therefore, the construction and validation of self-reports for the specific assessment of SAD is relevant. There are three self-reports of this type: the Separation Anxiety Assessment Scale (SAAS) [8], the Separation Anxiety Avoidance Inventory (SAAI) [9], and the Separation Anxiety Scale for Children (SASC) [10]. The SAAS has the disadvantage that its psychometric properties have not yet been published, whilst the SAAI focuses exclusively on avoidant behaviors, neglecting subjective aspects, such as distress and worry, both characteristic of SAD. Furthermore, these two self-reports assess separation anxiety in both childhood and adolescence at the same time (6–17 and 4–15 years, respectively), despite the fact that the symptoms vary with age. This is a problematic aspect, as it is inappropriate, for example, to present a four-year-old with item 2 of the SAAI, “Because I am anxious, I avoid being at home alone in the evening”, or a 17-year-old with item 4 of the SAAS, “How often are you afraid to be left at home with a babysitter?”

The SASC was developed for children aged 8 to 11 on the basis of the three-dimensional theory of anxiety [11], which, when applied to separation anxiety, postulates three inter-related components: a) cognitive, or worry that something bad will happen to the child and/or to his/her parents, b) psychophysiological, or distress resulting from the feelings of distress generated by excessive vegetative activation, and c) behavioral, or opposition to being separated from one's parents and/or away from home. The confirmatory factor analysis confirmed that the model of three correlated factors showed the best fit to the data, corroborating the factors Worry about separation and Distress from separation; however, contrary to what was expected, the third factor was not opposition, but rather Calm at separation. The child's calm at separation from parents can be interpreted a) cognitively, as the absence of worry – for example, I don't think my parents will have an accident or get sick; b) psychophysiologically, as the absence of distress – for example, I don't have stomach-ache or feel like crying; c) behaviorally, as the absence of opposition – for example, I don't do things to check whether my parents are OK or to try and be with them; d) positively, as the presence of calmness – for example, I feel calm/OK when my parents go away on a trip. The factor Calm at separation is problematic not only from the theoretical point of view, but also methodologically, as the internal consistency coefficient (Cronbach's alpha) was low (0.63). Moreover, the SASC has other methodological limitations: explained variance was just 32.80%, concurrent validity was calculated solely with two self-reports – one of anxiety and another of fears at school –, and neither its sensitivity nor its specificity was reported.

At a theoretical level it is interesting to explore whether opposition is a dimension of separation anxiety and whether calm is a positive factor that cannot be reduced to the mere absence of worry, distress and/or opposition. It is also important to have access to a psychometrically satisfactory instrument that addresses the deficiencies of the SASC – specifically, one that improves the construct validity by increasing the percentage of explained variance, that increases the convergent and discriminant validity on including measures of SAD, child anxiety, depression and anger, and that analyzes the structural validity by means of the sensitivity and specificity. Given the lack of a specific self-report rating scale for childhood SAD with adequate psychometric properties, which assesses the varied symptomatology of this disorder rather than focusing on just a single aspect of it, such as avoidant behavior, the general objectives of this study, taking as a starting point the SASC, were to develop (Study 1) and validate (Study 2) a self-report instrument, the Children's Separation Anxiety Scale (CSAS). In Study 1 we carry out an exploratory factor analysis with a bank of 40 items: the 26 from the original study [10] plus 14 new items from a pilot study. In Study 2 we calculate the validity, reliability, sensitivity and specificity of the CSAS.

## Study 1: Exploratory Factor Analysis

### Methods

#### Ethics Statement

The authors state that their research, approved by the Research Ethics Committee of the University of Murcia (Spain), has been performed in accordance with the ethical standards laid down in the 1964 Helsinki Declaration and its later amendments. The education authorities were informed of the study goals, and authorization was requested. Once such authorization had been obtained, the researchers interviewed the head teachers and the school counselors, informing them verbally and in writing about the aim of the study, so as to obtain their permission and encourage their cooperation. Finally, parents were informed by letter and requested to provide written consent for their children to participate in the study. The written parental consent was provided for all minors participating.

#### Participants

Random cluster sampling was carried out in two provinces in central and southern Spain, respectively. Primary units were provincial districts, secondary units were schools, and tertiary units were classrooms. We recruited 2,005 children from primary school grades 3 to 6 at 19 schools. A total of 97 (4.84%) were excluded due to errors or omissions in their responses, because their parents failed to provide informed consent, or because they were immigrants whose level of Spanish was too low. The sample was made up of 1,908 schoolchildren with a mean age of 9.61 (*SD* = 1.11). The chi-squared test for homogeneity of the distribution of frequencies indicated that there were no statistically significant differences between the eight groups of gender x age in Table 1 (χ^2^ = 0.48, *df* = 3, *p* = 0.92). Participants covered a wide range of socio-economic status, which was determined according to the school's type (public, grant-assisted private or private) and location (city, small town/village or village/rural).

**Table 1:** Exploratory factor analysis.

Item	Factor 1	Factor 2	Factor 3	Factor 4	Factor 5	Factor 6	Factor 7	Factor 8
28. Do you worry about something bad happening to you?	0.77	−0.04	0.11	−0.01	0.07	−0.01	0.01	0.06
21. Do you worry about something bad happening to your mom or dad?	0.76	0.01	0.03	0.05	0.04	0.10	0.11	0.02
25. Do you worry about your mom or dad having an accident?	0.74	−0.11	0.07	−0.01	0.05	−0.01	0.01	0.10
39. Do you worry about having an accident?	0.70	0.07	−0.02	0.00	−0.04	−0.03	−0.05	−0.13
31. Do you worry about getting sick?	0.67	0.10	0.03	0.14	0.15	0.13	0.17	−0.02
20. Do you worry about getting lost?	0.35	0.01	0.28	0.25	0.15	0.24	0.18	0.01
13. Do you worry about your mom or dad getting sick?	0.31	0.12	0.18	−0.13	−0.02	−0.08	−0.13	−0.04
26. Do you feel like crying when your mom or dad drops you off at school?	−0.05	0.76	0.11	0.04	0.08	0.09	0.06	0.01
23. Do you feel bad when your mom or dad drops you off at school?	0.02	0.70	0.19	0.04	0.04	0.09	0.04	0.09
15. Do you feel bad at school because your mom or dad isn't with you?	0.01	0.66	0.30	0.05	0.12	0.08	0.02	0.07
29. Are you afraid to eat at school in case you might vomit or choke?	0.08	0.62	0.01	0.06	0.16	0.03	0.26	0.05
16. Does your belly hurt when you have to leave your mom or dad?	−0.05	0.57	0.30	0.03	0.24	0.13	0.00	−0.10
32. Do you follow your mom or dad around the house?	0.03	0.34	0.19	0.02	0.09	0.34	0.04	0.06
12. Do you try to persuade your mom or dad not to go when he/she is planning to go on a trip?	0.08	0.20	0.66	0.07	0.02	0.03	0.24	0.05
18. Do you protest when your mom or dad plans to go out for the evening?	0.16	0.08	0.64	0.12	0.00	0.03	−0.05	−0.04
5. Do you protest when your mom or dad tells you he/she is going out?	0.12	0.08	0.60	0.05	0.15	−0.01	0.29	0.12
2. Do you try to call to your mom or dad when he/she isn't with you?	0.24	0.19	0.56	0.19	0.20	0.09	0.08	−0.01
14. Do you get sad, cry and protest when you're apart from your mom or dad?	−0.04	0.16	0.52	−0.01	0.08	0.13	0.10	0.06
6. Do you feel like crying when you're not with your mom or dad?	0.03	0.37	0.39	0.10	0.12	0.18	−0.01	−0.01
3. Do you get a headache when you have to leave your mom or dad?	−0.02	0.36	0.35	0.11	0.20	0.16	−0.04	−0.10
8. Do you worry that something bad will happen that stops you seeing your mom or dad again?	0.32	0.09	0.35	0.07	0.25	0.06	0.01	0.07
11. Are you calm when your mom or dad isn't with you?	0.01	0.02	0.19	0.66	0.06	−0.01	0.08	0.21
33. Are you calm when you're a long way from home?	−0.01	0.01	0.06	0.64	0.10	0.06	−0.01	0.16
27. Are you calm when you go away on a trip without your mom or dad?	0.05	0.04	−0.01	0.61	0.00	0.00	0.03	−0.07
17. Are you calm even if you can't call your mom or dad?	0.05	0.07	0.18	0.60	−0.01	−0.02	0.02	0.06
24. Are you calm when it gets dark and you're not with your mom or dad?	−0.01	0.04	−0.13	0.59	−0.01	0.04	−0.04	−0.06
1. Are you calm when your mom or dad goes away on a trip?	0.10	−0.02	0.31	0.37	−0.01	0.10	0.12	0.12
19. Do you have nightmares where something bad happens to your mom or dad?	0.08	0.22	0.15	0.06	0.75	0.06	0.07	0.08
7. Do you have nightmares where you're taken away from your mom or dad by force?	0.04	0.19	0.25	0.02	0.70	0.08	0.02	0.01
34. Do you have nightmares where something bad happens to you?	0.20	0.14	0.06	0.00	0.69	0.14	0.11	0.07
38. Do you ask your mom or dad to leave the light on when you go to bed at night?	0.01	0.22	0.10	−0.01	0.02	0.61	0.06	0.08
37. Are you frightened when you watch scary movies?	0.09	0.03	−0.01	0.00	0.13	0.55	0.19	−0.10
35. Are you afraid to stay at home alone?	0.13	0.21	0.29	0.07	0.10	0.49	0.16	0.02
4. Do you ask your mom or dad to sleep with you at night?	0.03	0.29	0.29	0.01	0.03	0.34	0.11	0.17
36. Do you refuse to go on camping trips?	−0.02	0.12	0.05	0.06	0.05	0.18	0.67	−0.04
40. Do you refuse to sleep away from home?	0.03	0.10	0.18	0.08	0.05	0.19	0.60	−0.02
22. Do you feel bad when your mom or dad leaves you at home with a babysitter?	0.17	0.10	0.27	−0.05	0.10	0.13	0.38	0.27
10. Are you calm when you get up in the mornings to go to school?	−0.04	0.09	−0.03	0.19	0.03	0.04	−0.01	0.70
30. Are you calm when you sleep alone in your bedroom?	−0.11	−0.05	0.09	0.18	0.11	0.40	−0.18	0.55
9. Do you think about going home to be with your mom or dad when you're at school?	0.11	0.36	0.04	−0.05	0.03	−0.17	0.17	0.44

#### Procedure

The researchers generated twenty new separation anxiety items that were evaluated with the same procedure as in the original study [10]. A pilot study was carried out with a random sample of 103 children aged 8 to 11 (*M* = 9.37, *SD* = 1.06) of both genders (54.43% girls). Six items were eliminated, a) at the suggestion of twelve experts in the psychopathology of development with broad clinical experience, and who acted as judges, b) because participants found them difficult to understand, and c) due to low item-test correlation. Examples of the eliminated items are “Do you refuse to sleep at a friend's house?” and “Do you forget your mom or dad when you go to an after-school activity?” The remaining 14 new items were added to the 26 original SASC items to make up the bank of 40 items employed in this study.

Participants responded to the bank of 40 separation anxiety items in the classroom and within normal class time. The researchers' assistants read the instructions aloud, provided individual help where necessary, and made sure that the pupils answered their own questions independently. In order to avoid bias, neither the assessors nor the participants were aware of the study's aims until the instruments had been handed in and processed.

#### Measure

We administered the bank of 40 separation anxiety items, made up of the 26 original items of the SASC [10] plus the 14 new items of the pilot study, which evaluates childhood separation anxiety by means of a 5-point Likert scale, ranging from 1 (never or almost never) to 5 (always or almost always).

#### Data Analysis

The underlying structure of the CSAS was identified by means of an iterative principal axis factor analysis with oblimin rotation because the factors were correlated. The data analysis was carried out with the SPSS statistics package, version 20.0.

### Results

The criteria for obtaining the factorial solution were: a) to retain factors with eigenvalues higher than 1 (Kaiser criterion), b) to assign to each factor the items that loaded higher than 0.40, and c) to include at least five items in each factor. Twenty items were removed: nine because the saturation was under 0.40 (items 1, 3, 4, 6, 8, 13, 20, 22, and 32) and eleven because the corresponding factor, which explained less than five per cent of the variance, included fewer than five items (items 7, 9, 10, 19, 30, 34, 35, 36, 37, 38, and 40). Four factors, each with five items, were obtained, explaining 46.91% of the variance. Factor 1, Worry about separation (items 21, 25, 28, 31, and 39), explained 13.91% of the variance. This is the cognitive component of anxiety, and refers to worry that something bad might happen to the child and/or to his/her parents. Factor 2, Distress from separation (items 15, 16, 23, 26, and 29), explains 12.32% of the variance. This is the psychophysiological component, which includes uncomfortable feelings, such as nausea or stomach ache, and the negative feelings separation can generate, such as wanting to cry. Factor 3, Opposition to separation (items 2, 5, 12, 14, and 18), explained 10.66% of the variance. This is the behavioral component, which refers to reactions for avoiding separation from the parents. Factor 4, Calm at separation (items 11, 17, 24, 27, and 33), explained 10.02% of the variance, and is the positive component, which reflects confidence in the child on being separated from his or her parents and/or on being away from home. Table 1 shows the CSAS factor structure.

## Study 2: Confirmatory Factor Analysis, Reliability and Validity

### Methods

#### Participants

In a similar way to Study 1, a random cluster sampling was carried out in two provinces of southeastern Spain. A total of 6,302 schoolchildren were recruited, from primary school grades 3 to 6, and 72 schools. In all, 286 (4.54%) were excluded for reasons similar to those reported in the previous study. Thus, the sample was made up of 6,016 children with a mean age of 9.59 (*SD* = 1.12). The chi-squared test for homogeneity of the distribution of frequencies highlighted the absence of statistically significant differences between the eight groups of gender x age in Table 2 (χ^2^ = 5.33, *df* = 3, *p* = 0.15). Socio-economic status of the participants was similar to that of those in Study 1.

**Table 2:** Child's gender and age.

	Age				
	8 (%)	9 (%)	10 (%)	11 (%)	Total (%)
*Study 1*					
Boys	212 (11.11)	233 (12.21)	250 (13.10)	275 (14.41)	970 (50.84)
Girls	200 (10.48)	222 (11.64)	255 (13.36)	261 (13.68)	938 (49.16)
Total	412 (21.59)	455 (23.85)	505 (26.47)	536 (28.09)	1,908 (100)
*Study 2*					
Boys	672 (11.17)	757 (12.58)	751 (12.48)	905 (15.04)	3,085 (51.28)
Girls	676 (11.24)	705 (11.72)	757 (12.58)	793 (13.18)	2,931 (48.72)
Total	1,348 (22.41)	1,462 (24,30)	1,508 (25,07)	1,698 (28,22)	6,016 (100)

Test-retest reliability was calculated with 1,926 children randomly selected from the sample, who responded to the CSAS again four weeks later. Diagnostic validity was calculated with 398 children also randomly selected from the sample, and who were assessed individually by means of a semi-structured interview based on the DSM-IV criteria.

Seventeen children were diagnosed with SAD through the ADIS-IV-C interview, accounting for 4.27%. The numbers of cases in the subsample used for this analysis (*n* = 398) were 8 children aged 8 (7.92%), 4 aged 9 (3.88%), 3 aged 10 (3.12%) and 2 aged 11 (2.04%). As regards the gender variable, 7 were boys (3.55%) and 10 were girls (4.98%).

#### Procedure

The processes of informing the education authorities, the head teachers and the parents, as well as those of requesting authorization and informed consent and administering the self-reports in the classroom, were similar to those described in Study 1.

With a view to avoiding too much disruption of the pupils' normal curriculum, and also to minimizing errors caused by fatigue, administration time of the self-reports was restricted to one hour. Thus, each participant responded, in the classroom situation, to just three instruments: I) the CSAS; II) one of the following, more extensive, self-reports (more than 30 items): the SAAS [8], the SCARED [6], the SCAS [7], or the State–Trait Anxiety Inventory for Children (STAIC) [12]; and III) one of the following, briefer, self-reports (less than 30 items): the Childhood Anxiety Sensitivity Index (CASI) [13], the School Fears Survey Scale (SFSS) [14], the Children's Depression Inventory (CDI) [15] or the State–Trait Anger Expression Inventory for Children and Adolescents (STAXI-CA) [16]. Thus, all participants filled out the CSAS, while each one of the eight self-reports was filled out by approximately a quarter of them (see *n* for each self-report in Table 3). The self-reports and the order of administration were assigned at random to each classroom group (20–25 pupils).

**Table 3:** Correlation coefficients of the CSAS with other self-reports.

	CSAS *n* = 6,016				
	1. Worry	2. Distress	3. Opposition	4. Calm	Total
SAAS *n* = 1,540					
Fear of being alone	0.13**	0.44**	0.39**	0.19**	0.46**
Fear of abandonment	0.18**	0.43**	0.39**	0.17**	0.52**
Somatic complaints/Fear of physical illness	0.20**	0.46**	0.36**	0.17**	0.56**
Worry about calamitous events	0.58**	0.24**	0.38**	0.22**	0.63**
Safety signals index	0.20**	0.52**	0.51**	0.24**	0.61**
Frequency of calamitous events	0.12**	0.18**	0.18**	0.10**	0.30**
Total	0.37**	0.54**	0.55**	0.27**	0.72**
SCARED *n* = 1,498					
Somatic/Panic	0.21**	0.48**	0.47**	0.19**	0.55**
Generalized anxiety	0.28**	0.32**	0.35**	0.16**	0.38**
Separation anxiety	0.32**	0.54**	0.55**	0.37**	0.62**
Social phobia	0.21**	0.29**	0.34**	0.14**	0.50**
School phobia	0.10**	0.45**	0.29**	0.16**	0.28**
Total	0.32**	0.54**	0.55**	0.24**	0.64**
SCAS *n* = 1,511					
Separation anxiety disorder	0.33**	0.41**	0.50**	0.38**	0.61**
Social phobia	0.23**	0.26**	0.28**	0.18**	0.41**
Obsessive/compulsive disorder	0.29**	0.32**	0.34**	0.20**	0.42**
Panic/Agoraphobia	0.18**	0.34**	0.34**	0.18**	0.38**
Physical injury fears	0.14**	0.22**	0.26**	0.16**	0.28**
Generalized anxiety disorder	0.32**	0.26**	0.32**	0.18**	0.44**
Total	0.34**	0.38**	0.44**	0.28**	0.55**
STAIC *n* = 1,467					
State	−0.01	0.27**	0.21**	0.22**	0.28**
Trait	0.19**	0.34**	0.37**	0.27**	0.42**
CASI *n* = 1,473	0.34**	0.41**	0.42**	0.23**	0.59**
SFSS *n* = 1,531	0.24**	0.26**	0.25**	0.16**	0.32**
CDI *n* = 1,527	0.11**	0.27**	0.22**	0.16**	0.27**
STAXI-CA *n* = 1,485					
State	−0.09**	0.07*	−0.07*	0.09**	−0.01
Trait	0.15**	0.17**	−0.03	0.10**	0.15**

*CSAS* Children's Separation Anxiety Scale, *SAAS* Separation Anxiety Assessment Scale, *SCARED* Screen for Child Anxiety Related Emotional Disorders, *SCAS* Spence Children's Anxiety Scale, *STAIC* State Trait Anxiety Inventory for Children, *CASI* Childhood Anxiety Sensitivity Index, *SFSS* School Fears Survey Schedule, *CDI* Children's Depression Inventory, *STAXI* State Trait Anger Expression Inventory.

*p≤0.05 ** *p*≤0.01.

#### Measures

With the aim of analyzing in detail the convergent and discriminant validity of the CSAS, we used a wide range of self-reports to assess variables related to separation anxiety.

CSAS. We administered the scale of 20 items resulting from Study 1, which assesses the frequency of separation anxiety symptoms on a 5-point scale: never or almost never (1), sometimes (2), often (3), very often (4), always or almost always (5).

SAAS [8]. We employed the Spanish translation made by the researchers, with the authors' permission, using the back-translation method [17]. It consists of 34 items whose response options are: never (1), sometimes (2), most of the time (3), and all the time (4). Total score on the scale is obtained by summing the four key symptom dimensions: fear of being alone, fear of abandonment, somatic complaints/fear of physical illness, and worry about calamitous events, plus the safety signals index and frequency of calamitous events (never, once, twice, three times or more). The subscales consist of 5 items, except for the safety signals index, which consists of 9 items. Internal consistency of the scale for the present study (Cronbach's alpha) was 0.88.

SCARED [6]. We applied the Spanish translation for children aged 8 to 12 [18]. The original instrument contains 41 items grouped in five subscales that assess different childhood anxiety disorders: somatic/panic (13 items), general anxiety (9 items), separation anxiety (8 items), social phobia (7 items), and school phobia (4 items). Symptom frequency is assessed by means of a three-point scale: 0 (almost never), 1 (sometimes), 2 (often). Internal consistency of the present sample was good (Cronbach's alpha  = 0.90).

SCAS [7]. We employed the Spanish adaptation for use with children aged 8 to 12 [19], which includes 38 items related to six childhood anxiety disorders: generalized anxiety disorder (6 items), panic attack/agoraphobia (9 items), social phobia (6 items), separation anxiety disorder (6 items), obsessive-compulsive disorder (6 items), and physical injury fears (5 items), plus 6 positive items that act as “fillers”, to offset the tendency to respond negatively. Frequency of each symptom is measured using a 4-point scale: never (0), sometimes (1), often (2), always (3). Internal consistency (Cronbach's alpha) of the complete scale in the present study was 0.88.

STAIC [12]. We applied the Spanish adaptation for children and adolescents aged 9 to 15 [20]. This instrument is one of the most well studied and commonly used general anxiety self-report rating scales. It is composed of two 20-item subscales with three response alternatives (1 =  rarely, 2 =  sometimes, 3 =  often), which evaluate current level of anxiety (state) and chronic symptoms of anxiety (trait). Internal consistency (Cronbach's alpha) for the present sample was 0.87 for both state anxiety and trait anxiety.

CASI [13]. We applied the Spanish adaptation for children aged 9 to 11 [21]. Sensitivity to anxiety is the fear of the anxiety symptoms produced by the belief that the feelings of anxiety are dangerous or harmful. It is considered to predispose the individual to the development of anxiety disorders. The instrument consists of 18 items for assessing this risk factor with a 3-point Likert-type scale (1 =  none, 2 =  some, 3 =  a lot), e.g. “It scares me when I feel like I am going to throw up”. The internal consistency coefficient for the sample in our study was 0.85.

SFSS [14]. This was designed for children aged 8 to 11. The 25 items on school fears are assessed via a three-point scale: 0 (not at all), 1 (a little), 2 (a lot). The scale includes fears related to SAD, such as “Separating from parents to go to school”, and others unrelated to the disorder, such as “Getting bad exam marks”. Persistent refusal to go to school because of fear of separation is one of the characteristics of SAD. Internal consistency of this instrument was good (α = 0.90).

CDI [15]. We used the Spanish adaptation for children and adolescents aged 7 to 15 [22]. Depressed mood is often associated with SAD. The CDI is the self-report most widely used for assessing depressive symptomatology in childhood. It consists of 27 items with three response options, and the child must choose from them the one that best describes him or her in the last two weeks. For the present sample the internal consistency was very high (Cronbach's alpha  = 0.95).

STAXI-CA. Del Barrio and Aluja [16] adapted the STAXI-2 [23] for children and adolescents aged 8 to 17. This instrument consists of 32 items in four 8-item subscales: state anger, trait anger, expression of anger, and control of anger. The child marks the option that best describes him/her: 1 (a little), 2 (quite a lot), 3 (a lot). In our study we used only the subscales state anger (α = 0.93) and trait anger (α = 0.80). The relation with separation anxiety is expected to be lower than with state anxiety and with trait anxiety.

*Anxiety Disorders Interview Schedule for Children for DSM-IV* (ADIS-IV-C) [24], Spanish adaptation [25]. This is a semi-structured interview, based on the diagnostic criteria of the DSM-IV, and which is widely used with children and adolescents aged 7 to 17. It contains modules on all anxiety disorders, including SAD and school refusal, as well as dysthymia, major depressive disorder, attention-deficit/hyperactivity disorder, conduct disorder, and oppositional-defiant disorder. Each diagnosis is completed with a severity assessment made by the clinician using a nine-point scale, from 0 (none) to 8 (very severely disturbing/disabling). Twenty-five psychologists with Masters qualifications in child psychopathology and clinical psychology received intensive training in the use of the ADIS-IV-C with the help of a specific manual [26]. In the present sample, the kappa coefficient obtained for SAD was 1 (perfect agreement).

#### Data Analysis

The structure of the CSAS obtained in Study 1 was examined by means of confirmatory factor analysis. Internal consistency was calculated with Cronbach's alpha coefficient, and a classical item analysis was carried out to obtain the correlations of the items with the corresponding factor and with the CSAS total score. Concurrent validity and test-retest reliability were calculated with the Pearson product-moment correlation coefficient. Sensitivity and specificity were studied by means of a receiver operating characteristic (ROC) curve and area under the curve (AUC). A 2×4 between-subjects analysis of variance was carried out for examining the differences by gender and age in separation anxiety. The analyses were carried out using the statistics packages SPSS version 20.0, AMOS version 20.0 and MedCalc version 12.5.

### Results

#### Gender and Age Differences

In the total sample we found a significant decrease in separation anxiety with age (*F*_3, 6012_ = 49.01, *p*<0.001). Mean score on the CSAS was 59.02 (*SD* = 11.07) at age 8, 56.72 (*SD* = 12.34) at age 9, 54.63 (*SD* = 11.77) at age 10 and 49.84 (*SD* = 11.73) at age 11. Magnitude of the differences was large between the children 8 aged and 11 (*d* = 0.80), moderate between those aged 9 and 11 (*d* = 0.57), and small in the remaining cases (*d*<0.50).

Girls' mean score (*M* = 56.08, *SD* = 12.35) was significantly higher than that of boys (*M* = 53.49, *SD* = 11.98), though the difference was small (*F*_1, 6014_ = 12.37, *p*<0.001, *d* = 0.21). There was no interaction between the gender and age variables (*F*_3, 6012_ = 0.47, *p* = 0.71).

#### Confirmatory Factor Analysis

Four alternative models were assessed: 1) the null or independent model (M_0_); 2) the one-factor model (M_1_), in which the 20 scale items were forced to load in a general separation anxiety factor; 3) the uncorrelated four-factor model (M_4_); and 4) the four correlated factors model (M_4*_).

To examine the adequacy of the assessed models we used six fit indexes: the Root Mean Square Error of Approximation (RMSEA), the Goodness of Fit Index (GFI), the Adjusted Goodness of Fit Index (AGFI), the Normed Fit Index (NFI), the Comparative Fit Index (CFI), and the Tucker-Lewis Index (TLI), as well as the chi-square statistic (χ^2^). Browne and Cudeck [27] recommend a value of less than 0.05 for RMSEA. Hu and Bentler [28] suggest 0.95 for the NFI, CFI and TLI, as well as using a combination of fit indexes in order to reduce both type I and II errors. For GFI and AGFI, values above 0.90 are considered acceptable (Table 4).

**Table 4:** Fit indexes for confirmatory factor models.

Model	χ^2^	*df*	*p*	RMSEA	GFI	AGFI	NFI	CFI	TLI
M_0_	16627.45	190	0.00	—	—	—	—	—	—
M_1_	15617.79	170	0.00	0.11	0.77	0.72	0.52	0.52	0.46
M_4_	3332.27	170	0.00	0.07	0.92	0.90	0.80	0.81	0.80
M_4*_	1347.58	164	0.00	0.04	0.97	0.96	0.93	0.93	0.92

Four correlated factors model; χ^2^ Chi-Square test, *df* Degrees of freedom, *p* Probability, *RMSEA* Root Mean Square Error of Approximation, *GFI* Goodness of Fit Index, *AGFI* Adjusted Goodness of Fit Index, *NFI* Normed Fit Index, *CFI* Comparative Fit Index, *TLI* Tucker-Lewis Index.

The chi-square statistic was significant, demonstrating a poor fit for all the models. However, these values must be considered with care, since the goodness of fit statistic χ^2^ depends on the sample size. This statistic is very powerful with large samples, and can detect significant differences in spite of the fact that the models fit the data well; therefore, we took into account other fit indexes. The best fit of the models studied was shown by the four correlated factors model, which gave acceptable values for RMSEA GFI, AGFI, NFI, CFI and TLI. Table 5 shows the correlation coefficients among factors and with the total score of the CSAS.

**Table 5:** Correlation matrix among factors and with CSAS total score.

	1. Worry	2. Distress	3. Opposition	4. Calm	Total
1. Worry	----	----	----	----	----
2. Distress	0.21*	----	----	----	----
3. Opposition	0.27*	0.51*	----	----	----
4. Calm	0.12*	0.30*	0.37*	----	----
Total	0.60*	0.65*	0.77*	0.70*	----

*p<0.001.

#### Internal Consistency and Item Analysis

The internal consistency coefficients (Cronbach's alpha) were 0.82 for the CSAS, 0.83 for Factor 1, Worry about separation, 0.76 for Factor 2, Distress from separation, 0.72 for Factor 3, Opposition to separation, and 0.75 for Factor 4, Calm at separation. The item-subscale correlations were acceptable, with a range of 0.57 to 0.79. All the items obtained an item-test correlation higher than 0.30, indicating their adequate behavior. Table 6 shows the item-subscale correlation (IS-R), the corrected item-subscale correlation (IS-R_c_), the item-test correlation (IT-R), the corrected item-test correlation (IT-R_c_), the mean (M), and the standard deviation (SD) of the 20 CSAS items.

**Table 6:** Item analysis of CSAS.

Items	IS-R	IS-R_C_	IT-R	IT-R_C_	M	SD
Factor 1: Worry about separation						
21	0.79	0.60	0.45	0.34	4.33	1.19
28	0.75	0.63	0.45	0.36	4.01	1.43
31	0.75	0.49	0.33	0.24	4.24	1.27
25	0.73	0.58	0.38	0.28	4.42	1.17
39	0.68	0.54	0.46	0.44	3.69	1.55
Factor 2: Distress from separation						
15	0.72	0.53	0.51	0.41	1.51	1.06
23	0.72	0.53	0.47	0.36	1.44	1.04
26	0.71	0.53	0.43	0.31	1.34	0.97
29	0.66	0.37	0.50	0.31	1.76	1.35
16	0.63	0.42	0.41	0.32	1.42	1.01
Factor 3: Opposition to separation						
12	0.72	0.49	0.52	0.44	2.14	1.54
14	0.70	0.46	0.63	0.52	2.73	1.53
18	0.68	0.44	0.49	0.42	2.22	1.51
2	0.63	0.36	0.45	0.36	2.52	1.51
5	0.57	0.36	0.39	0.30	1.68	1.15
Factor 4: Calm at separation						
11	0.69	0.42	0.43	0.35	2.92	1.60
17	0.64	0.39	0.37	0.33	3.09	1.60
33	0.63	0.38	0.35	0.28	2.88	1.60
27	0.62	0.35	0.31	0.24	3.21	1.61
24	0.58	0.37	0.31	0.23	3.18	1.64

*IS-R* Item-scale correlation, *IS-R_c_* Corrected correlation item-scale, *IT-R* Item-test correlation, *IT-R_c_* Corrected correlation item-test, *M* Mean, *SD* Standard deviation.

#### Test-retest Reliability

Test-retest reliability coefficients were *r* = 0.83 for CSAS, *r* = 0.69 for Worry about separation, *r* = 0.67 for Distress from separation, *r* = 0.70 for Opposition to separation, and *r* = 0.66 for Calm at separation.

#### Convergent and Discriminant Validity

Table 3 shows the correlation coefficients of the factors and the total CSAS score with other self-reports. Correlation of the CSAS total score with other measures of separation anxiety, specifically with the SAAS total score and with the score on the corresponding subscales of the SCARED and the SCAS were high, ranging between 0.61 and 0.72.

Analysis of the correlation coefficients of the CSAS and SAAS also reveals a close relationship between the similar factors of the two instruments, Worry about separation (CSAS) and Worry about calamitous events (SAAS) (*r* = 0.58), Distress from separation (CSAS) and Somatic complaints/Fear of physical illness (SAAS) (*r* = 0.46), and Opposition to separation (CSAS) and Safety Signals Index (SAAS) (*r* = 0.51). Correlations of the Calm at separation (CSAS) factor, with no equivalent in the SAAS, were low with all the factors of the latter scale (*r*<0.25).

Correlation of CSAS total score was good with anxiety sensitivity, higher with trait anxiety than with state anxiety, weak with school fears and depression, very low with trait anger, and non-existent with state anger.

#### Sensitivity and Specificity

Sensitivity was operationalized as the percentage of children with a SAD diagnosis according to the ADIS-IV-C who were correctly classified using the CSAS total score. Specificity was operationalized as the percentage of children that did not receive a SAD diagnosis in the interview and were correctly identified by the CSAS. The inverse relation between sensitivity and specificity requires equilibrium between the two for selecting the optimum cut-off point. For determining the positive predictive value (PPV) we calculated for each cut-off point the percentage of children with SAD who actually met the diagnostic criteria for this disorder. For determining the negative predictive value (NPV) we calculated for each cut-off point the percentage of children without SAD who actually did not meet the diagnostic criteria for this disorder. A receiver operating characteristic (ROC) curve and area under curve (AUC) were analyzed to establish the optimal cut-off score. The results showed that the AUC for ROC for the cut-off of 68 was 0.96 (95% CI, 0.93–0.98), and was significant versus chance or a random ROC line (*p*<0.0001). This suggests that there is a 96% probability of a child with SAD scoring higher on the CSAS than children without SAD.

**Figure 1:**
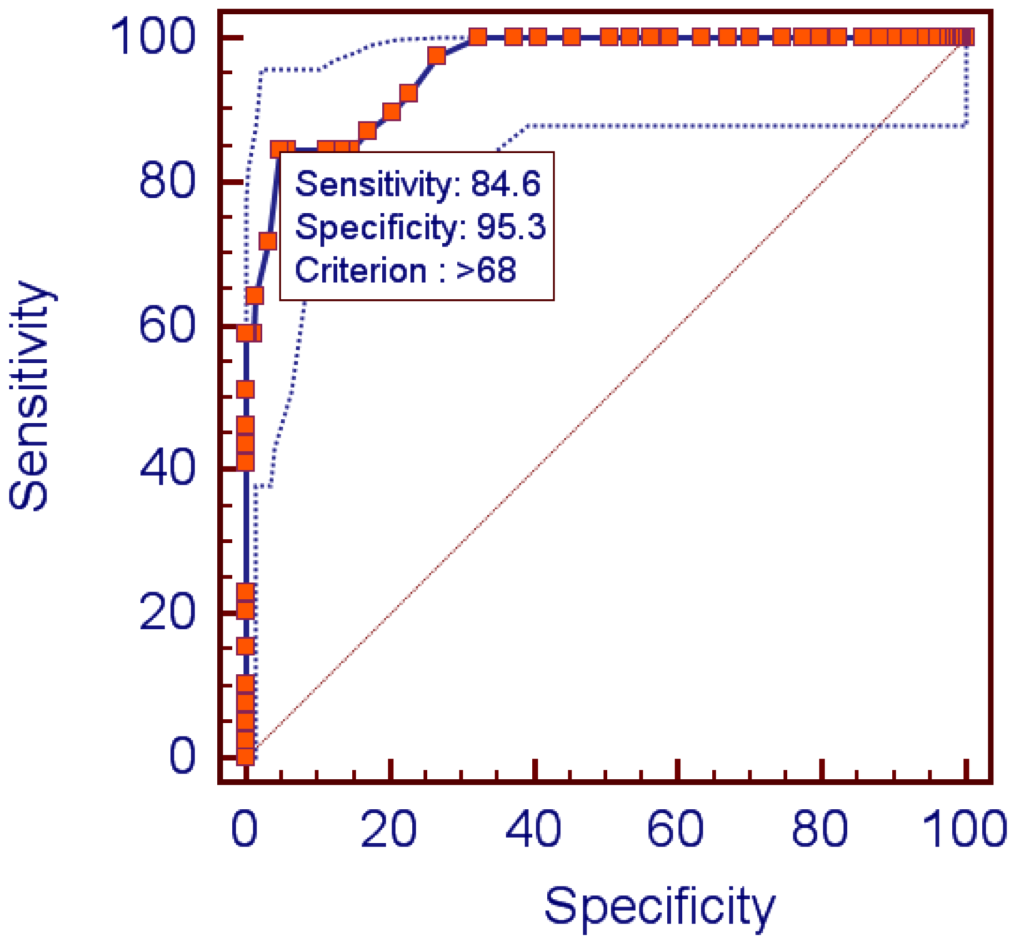
ROC curves of the CSAS.

In order to assess the global diagnostic effectiveness we calculated the Youden Index [29], which is the maximum vertical distance or the difference between the ROC curve and the diagonal or chance line. The results revealed that a score of 68 in the CSAS is the optimal cut-off, because it achieved the best balance, with good sensitivity (85%, 95% CI, 70–94) and specificity (95%, 95% CI, 92–97), a PPV of 76 and an NPV of 98. The Youden Index was 0.80 (Fig. 1).

## Discussion

The CSAS comprises 20 items grouped in four factors: Worry about separation, Distress from separation, Opposition to separation, and Calm at separation. In accordance with the three-dimensional theory [11], the three first factors correspond to the three response systems of anxiety, and permit assessment of the symptomatology of SAD that is, excessive and persistent worry about loss of or harm to attachment figures or the child him/herself, excessive and recurrent distress in the child on being separated from attachment figures, and resistance to or refusal to accept separation from attachment figures. In contrast, Calm at separation is a novel factor. Clinical psychologists expert in this field, who judged the pertinence of the item bank in the original study [10] with a view to controlling the tendency to respond negatively, suggested the items of this subscale. It was expected that on inverting the scores on the items they would distribute themselves among the other factors according to whether the anxiety was of a cognitive (worry), psychophysiological (distress) or behavioral (opposition) nature. However, there emerged the Calm at separation factor, a positive dimension that reflects child's self-confidence in situations of separation. In some studies with adolescent population [30], [31] on Personal Report of Confidence as Speaker [32] it was found that confidence was not equivalent to a low level or absence of fear, but rather to self-confidence that makes public-speaking a reinforcing activity (for example, “I face the prospect of making a speech with complete confidence” or “At the conclusion of a speech I feel that I have had a pleasant experience”). Likewise, children differ not only in their level of separation anxiety, but also in their degree of security and enjoyment when they are home alone or they go away on a trip without their parents. The CSAS is the only self-report that includes a positive dimension, and it would be interesting to study the role of this dimension as a protection factor against SAD.

The Frequency of calamitous events of the SAAS shows the lowest correlations with the CSAS factors, since these events refer not to clinical characteristics, but to situations that trigger SAD. On the other hand, the Safety signals index, which is not considered a key symptom dimension, can be understood as distress from or opposition to separation situations. The two SAAI factors, Going to school, to bed alone (4 items) and Being or going home alone when no-one is there (3 items) refer to the avoidance of different separation situations. A study carried out with 931 parents of children aged 3 to 5 for evaluating early-onset separation anxiety found, together with a subjective fear and worry factor, two factors of avoidance, one related to sleeping – for example, “If your child wakes up during the night, does he/she call you insistently so that you have to go to his/her bedroom and calm him/her down? – and another related to everyday events – for example, “If you have to attend a meeting and leave your child with a neighbor or friend for a few hours, does your child try to resist?” [33], [34]. Future research should explore whether the first-order behavioral factor opposition/avoidance groups second-order factors defined by different separation situations: night, school, home alone, etc.

Internal consistency was good for the CSAS and for the Worry about separation factor and adequate for the remaining factors. Although the values are not high, they should be interpreted taking into account that the factors are made up of just five items. These coefficients are similar to or higher than those found for such short scales; thus, the internal consistency obtained with Cronbach's alpha for the six-item factor Separation anxiety disorder of the SCAS was 0.70, 0.62 and 0.59 for school samples from Australia [35], Belgium [36] and Spain, respectively [19]. Temporal stability, with a test-retest interval of four weeks, was also good for the CSAS and adequate for the factors.

Correlation between the Worry about separation factor of the CSAS and the separation anxiety and generalized anxiety subscales of the SCAS and SCARED are similar. This can be explained, on the one hand, by the high level of comorbidity of childhood anxiety disorders [37] and, on the other, by the fact that worry (e.g., about family members' health) is an element common to separation anxiety disorder and generalized anxiety disorder. Correlation with the SFSS is not high. There is a long tradition that associates school refusal and SAD. In 1932, Broadwin [38] distinguished truancy from rejection to attend school out of fear that something will happen to one's mother – or she might even die – if the child is not at home. However, only 22.2% of children and adolescents with SAD refuse to go to school due to fear of separation [39]. Schoolchildren are more afraid of failure and punishment at school than of being separated from their parents to go to school [14]. Correlation with the CDI is explained by the substantial comorbidity of the SAD with childhood major depressive disorder (37%) [40]. The fact that the correlation of the CSAS with the measures of anxiety, especially with those of separation anxiety, is on the whole markedly greater than that with the CDI, reveals that the scale is an indicator of symptoms of anxiety, mainly of separation anxiety, more than depression. The finding that the relation with trait anger is weak, and with state anger is non-existent, also supports the discriminant validity of the CSAS.

The present study has two important limitations. First, the school sample recruited restricts generalization of the results, and second, the only source of information used was the child him/herself. Future studies should analyze the psychometric properties of the CSAS with clinical samples and validate the version for parents, since SAD is a relational anxiety disorder – that is, it also involves major attachment figures, so that multi-source assessment of this childhood disorder is especially relevant.

**Figure 2:**
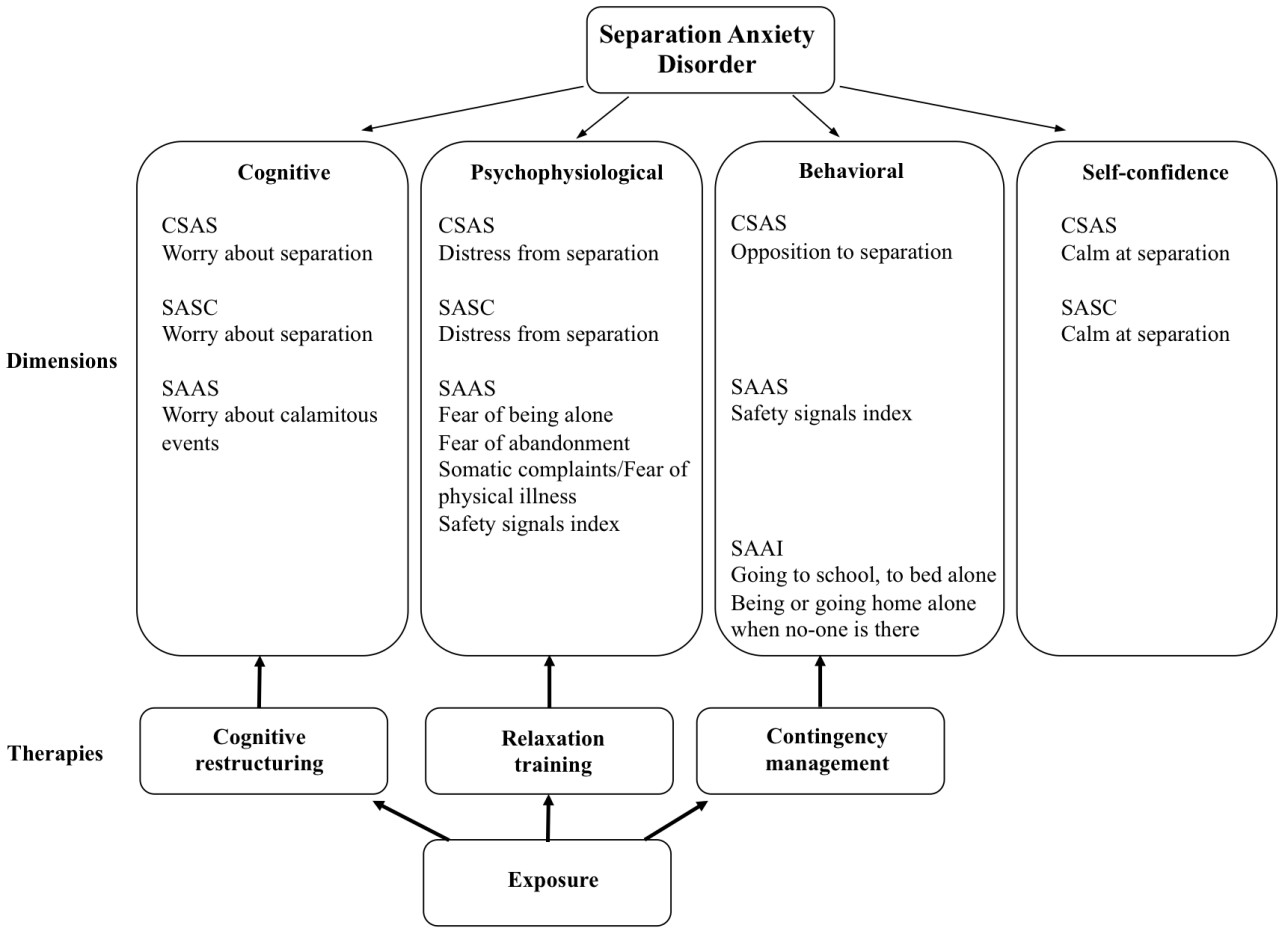
Assessment and treatment of SAD.

In sum, the CSAS is a self-report that assesses the varied symptomatology of SAD, including the positive dimension Calm at separation, with good internal consistency, high temporal stability, adequate convergent and discriminant validity, and good sensitivity and specificity. It is an instrument that can be of great utility in the framework of multi-method assessment. Moreover, if it can be shown with clinical population that the psychometric properties are similar, the CSAS would constitute a helpful tool in the context of diagnosis and treatment planning. Although the focus of therapy for SAD is exposure, it is recommended that it be complemented with other therapeutic procedures so as to facilitate implementation, increase therapeutic collaboration and prevent relapse. Coping Cat [41], a pioneering program in the treatment of childhood anxiety disorders, including SAD, facilitates exposure with various therapeutic strategies taught to children using the acronym FEAR: *F*eeling frightened? (Relaxation), *E*xpecting bad things to happen? (Cognitive restructuring), *A*ctions and attitudes than can help (Problem-solving), *R*esults and rewards (Self-reinforcement). Similarly, the FRIENDS for Life program [42] uses the acronym FRIENDS: *F*eeling worried? *R*elax and feel good, *I*nner thoughts, *E*xplore plans, *N*ice work, so reward yourself, *D*on't forget to practice, *S*tay calm, you know how to cope now. As regards parents, they are taught to reinforce separation behaviors and extinguish their children's complaints. Thus, the CSAS could guide selection of the coping skills and family intervention to be applied – that is, cognitive restructuring if the SAD profile is worry, relaxation training if it is distress, and contingency management if it is opposition (see Fig. 2).
